# Prognostic implications of soluble programmed death-ligand 1 and its dynamics during chemotherapy in unresectable pancreatic cancer

**DOI:** 10.1038/s41598-019-47330-1

**Published:** 2019-07-31

**Authors:** Hyunkyung Park, Ju-Hee Bang, Ah-Rong Nam, Ji Eun Park, Mei Hua Jin, Yung-Jue Bang, Do-Youn Oh

**Affiliations:** 1 Department of Internal Medicine, Seoul National University Hospital, Seoul, Korea; 2 Cancer Research Institute, Seoul National University College of Medicine, Seoul, Korea

**Keywords:** Tumour biomarkers, Tumour immunology

## Abstract

In pancreatic cancer, acquiring a sufficient amount of tumor tissue is an obstacle. The soluble form of PD-L1 (sPD-L1) may have immunosuppressive activity. Here, we evaluated the prognostic implications of sPD-L1 in unresectable pancreatic cancer. We prospectively enrolled 60 patients treated with first-line FOLFIRINOX chemotherapy. We collected blood samples at diagnosis, first response assessment and disease progression. Serum sPD-L1 levels were measured using enzyme-linked immunosorbent assays. The median sPD-L1 level was 1.7 ng/mL (range, 0.4–5.7 ng/mL). Patients with low sPD-L1 level (<4.6 ng/mL) at diagnosis showed better overall survival (OS) than those with high sPD-L1 level (*P* = 0.015). Multivariate analysis identified sPD-L1 and the neutrophil-to-lymphocyte ratio as independent prognostic factors for OS. During chemotherapy, more patients achieved complete response (CR)/partial response (PR) as their best response when sPD-L1 was decreased at the first response assessment (*P* = 0.038). In the patients who achieved CR/PR as their best response, sPD-L1 was significantly higher at the time of disease progression than at the first response assessment (*P* = 0.025). In conclusion, the sPD-L1 level at diagnosis exhibits a prognostic value in pancreatic cancer. Furthermore, sPD-L1 dynamics correlate with disease course and could be used to understand various changes in the tumor microenvironment during chemotherapy.

## Introduction

With recent advances in immuno-oncology, many biomarkers of immune checkpoints have received increasing attention due to their roles as prognostic factors and therapeutic targets. Biomarkers such as cytotoxic T-lymphocyte-associated antigen 4 (CTLA-4) ligands, interleukin-10, transforming growth factor β (TGF-β), and programmed death-ligand 1 (PD-L1) are well-known molecules involved in escape from host immune surveillance and are associated with cancer cell proliferation^1,2^. In particular, PD-L1 is the most promising immune checkpoint molecule used as a potential target for immunotherapy.

PD-L1, a B7 superfamily member, is expressed in tumor cells and binds to programmed death-1 (PD-1) on T cells. After the PD-L1/PD-1 interaction occurs, inhibitory signals are transmitted to T cells, thereby suppressing T cell proliferation and reducing cytokine secretion^1^. Tumor cells are then able to escape immune recognition and the host immune response. In various cancers, such as lung cancer, breast cancer, and renal cell carcinoma, overexpression of PD-L1 in tumor cells results in aggressive behavior and is associated with a poor prognosis^3–5^. Additionally, PD-L1 expression can predict treatment response in anti-PD-1/PD-L1 agent-treated patients^6^. Therefore, an evaluation of PD-L1 expression could predict patient prognosis and treatment response in certain types of cancer.

A biopsy is needed to evaluate PD-L1 expression in tumor tissue and the tumor microenvironment, which is the primary limitation of this method. However, soluble serum biomarkers have been regarded as promising surrogates because they can reflect the tumor status and predict survival outcomes through a minimally invasive modality^7,8^. Therefore, the use of soluble markers could help in understanding the dynamics of the interaction between host immune responses and tumor cells or the tumor microenvironment.

The soluble form of PD-L1 (sPD-L1) has been reported to have prognostic value in various cancers, including lymphoma, gastric cancer, and lung cancer^7–9^. In addition, a few studies have recently reported the dynamics of sPD-L1 in cancer patients during chemotherapy treatment^8^.

Despite previous study results, in pancreatic cancer, the prognostic role of the PD-L1/PD-1 signaling axis remains uncertain. Some studies confirmed the prognostic importance of the PD-L1/PD-1 signaling axis in pancreatic cancer^10–12^. However, the results of an early trial of a monotherapy checkpoint blockade approach were disappointing and suggested that evaluating the immune checkpoint alone may be insufficient to explain the poor prognosis of pancreatic cancer^13^. Therefore, the role of PD-L1/PD-1 in pancreatic cancer requires further investigation, especially because in many cases, it is difficult to acquire a sufficient amount of tumor tissue to evaluate PD-L1 expression, and the value of sPD-L1 has not yet been studied.

The aims of this study were to measure serum sPD-L1 levels in unresectable pancreatic cancer patients who were treated with palliative first-line chemotherapy and to evaluate the prognostic role of sPD-L1 as well as its dynamics during chemotherapy.

## Methods

### Patient characteristics

We prospectively recruited pathologically confirmed unresectable pancreatic cancer patients who were treated with FOLFIRINOX as their first-line palliative chemotherapy regimen. Between 2013 and 2015, 60 patients who provided informed consent for the biomarker analysis study at Seoul National University Hospital were enrolled; all were ethnically Korean. FOLFIRINOX chemotherapy consisted of oxaliplatin (85 mg/m^2^ on day 1), irinotecan (180 mg/m^2^ on day 1), leucovorin (400 mg/m^2^ on day 1), fluorouracil (5-FU; 400 mg/m^2^ on day 1), and a continuous infusion of 5-FU (2,400 mg/m^2^ on day 1 over 48 hours). Clinical data were collected by reviewing medical records that included demographic information and the results of laboratory exams, including total bilirubin, albumin, and cancer antigen 19-9 (CA 19-9) levels and blood cell counts (neutrophil, lymphocyte, and platelet counts). The neutrophil-to-lymphocyte ratio (NLR) and the platelet-to-lymphocyte ratio (PLR) were calculated by dividing the neutrophil or platelet count, respectively, by the lymphocyte count. The results of a laboratory test performed at the time of diagnosis with unresectable pancreatic cancer were used in the analysis. We evaluated the disease state during FOLFIRINOX chemotherapy by computed tomography examination every three chemotherapy cycles. Response assessment was performed according to the Response Evaluation Criteria in Solid Tumors (RECIST) criteria version 1.1^14^. The best overall response rate (ORR) was defined as the proportion of patients who achieved a complete response (CR) or partial response (PR) as their overall response during chemotherapy^15^.

### Measurement of sPD-L1 levels

We prospectively collected blood samples from patients at the time of diagnosis (prechemotherapy), at the first response assessment (postchemotherapy, after three cycles of chemotherapy), and at the time of disease progression. Serum sPD-L1 levels were measured using an enzyme-linked immunosorbent assay **(**ELISA; PDCD1LG1 ELISA kit, USCN Life Science) according to the manufacturer’s instructions^16^. Each sample was analyzed in duplicate.

### Statistical analysis

Fisher’s exact test or Pearson’s chi-squared test was used to compare categorical variables. The comparison of continuous variables was performed using an independent or paired *t*-test or one-way ANOVA, as appropriate. Progression-free survival (PFS) and overall survival (OS) were estimated using the Kaplan-Meier method. PFS was defined as the time from the date of initiation of FOLFIRINOX chemotherapy to the date of disease progression. OS was defined as the time from the date of initiation of FOLFIRINOX chemotherapy to the date of either death or last follow-up. A receiver operating characteristics (ROC) curve was used to determine the cut-off values of the NLR, the PLR, and sPD-L1 levels to best predict survival. The cut-off values used for albumin, total bilirubin, and CA 19-9 levels were the corresponding normal values. Clinical variables with univariate *P*-values < 0.2 were considered for inclusion in multivariate analyses, which were performed using the logistic regression model or Cox proportional hazard model, as appropriate. All statistical tests were two-sided, and a statistically significant difference was defined as *P* < 0.05. Statistical analyses were performed using IBM SPSS version 23.0 (IBM, Armonk, NY, USA).

### Ethical considerations

This study was approved by the institutional review board at Seoul National University Hospital (IRB; H-1307-146-507) and was conducted in accordance with the guidelines of the Declaration of Helsinki for biomedical research. Informed consent was obtained from all participants.

## Results

### Patient characteristics

The mean and median values of the sPD-L1 level at initial diagnosis (prechemotherapy) (n = 60) were 2.2 and 1.7 ng/mL (range, 0.4–5.7 ng/mL), respectively. ROC curve analysis was used to determine the cut-off value of sPD-L1, and the cut-off value of 4.6 ng/mL achieved the highest combination of sensitivity and specificity for the prediction of OS.

Baseline characteristics of the patients are summarized in Table 1, and the patients were divided into two groups according to their sPD-L1 levels. No statistical differences in the sPD-L1 level were observed when patients were stratified by different clinical characteristics, including age, sex, disease extent, CA 19-9 level, total bilirubin level, albumin level, NLR value, and PLR value (all *P* > 0.05).

**Table 1:** Baseline characteristics of patients stratified according to the sPD-L1 level.

Variable		sPD-L1 < 4.6 ng/mL (*N* = 52)	sPDL1 ≥ 4.6 ng/mL (*N* = 8)	*P*-value
Age, years	≥60	23 (44.2)	2 (25.0)	0.449
	<60	29 (55.8)	6 (75.0)	
Sex	Male	31 (59.6)	2 (25.0)	0.124
	Female	21 (40.4)	6 (75.0)	
Disease extent	Locally advanced	13 (25.0)	1 (12.5)	0.667
	Metastatic	39 (75.0)	7 (87.5)	
CA19-9, U/mL	Elevated (≥37.0)	39 (75.0)	8 (100.0)	0.182
	Decreased (<37.0)	13 (25.0)	0 (0.0)	
Total bilirubin, mg/dL	Elevated (>1.2)	12 (23.1)	0 (0.0)	0.338
	Normal (≤1.2)	40 (76.9)	8 (100.0)	
Albumin, g/dL	Normal (≥3.3)	49 (94.2)	8 (100.0)	1.000
	Decreased (<3.3)	3 (5.8)	0 (0.0)	
NLR	Increased (≥1.83)	41 (78.8)	5 (62.5)	0.374
	Decreased (<1.83)	11 (21.2)	3 (37.5)	
NLR	Mean	3.00 (±1.55)	2.97 (±1.70)	0.940
PLR	Increased (≥109.6)	37 (71.2)	6 (75.0)	1.000
	Decreased (<109.6)	15 (28.8)	2 (25.0)	
PLR	Mean	156.6 (±60.01)	171.8 (±72.55)	0.388

Abbreviations: cancer antigen 19-9 = CA 19-9; NLR = neutrophil-to-lymphocyte ratio; PLR = platelet-to-lymphocyte ratio; and sPD-L1 = soluble programmed death-ligand 1.

Patient responses to FOLFIRINOX chemotherapy during the follow-up period were as follows: 2/60 (3.3%) patients achieved a CR; 20/60 (33.3%) patients achieved a PR; 30/60 (50%) patients achieved stable disease (SD); and 8/60 (13.3%) patients exhibited progressive disease (PD). Twenty-two (36.7%) patients achieved a CR or PR as their overall response during chemotherapy.

### Survival outcomes

The median follow-up duration of the 60 patients was 11.4 months (95% confidence interval [CI], 6.9–14.8 months), and the median PFS and OS were 6.5 (95% CI, 4.9–8.1 months) and 10.3 (95% CI, 8.5–12.1 months) months, respectively.

**Figure 1:**
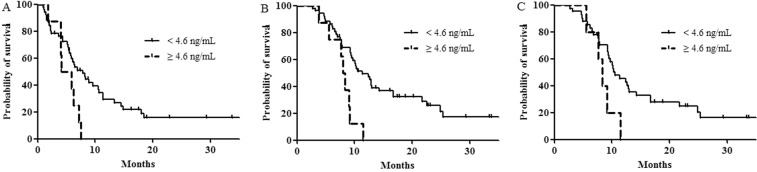
Survival outcomes. (**A**) Progression-free survival of patients stratified according to soluble programmed death-ligand 1 (sPD-L1) levels (median 4.1 *vs*. 7.8 months, *P* = 0.021). (**B**) Overall survival of patients stratified according to sPD-L1 levels (median 8.0 *vs*. 12.6 months, *P* = 0.003). (**C**) Overall survival of metastatic pancreatic cancer patients stratified according to sPD-L1 levels (median 8.4 months *vs*. 10.2 months, *P* = 0.028).

In a univariate analysis for PFS, older age (≥60 years), a low sPD-L1 level (<4.6 ng/mL), a low NLR (<1.83), and a low PLR (<109.6) were associated with prolonged PFS (Table 2; Fig. 1A). In a multivariate analysis, age, the NLR, the PLR, and sPD-L1 were no longer significantly associated with prolonged PFS, although a high NLR (hazard ratio [HR], 3.141, *P* = 0.061) and high sPD-L1 level (HR, 2.077, *P* = 0.080) showed a trend toward worse PFS.

**Table 2:** Univariate and multivariate Cox regression analyses for progression-free survival.

Variable		Univariate analysis		Multivariate analysis		
		mPFS (95% CI) (months)	*P*-value	HR	95% CI	*P*-value
Age, years	≥60	10.5 (7.0–14.0)	0.026	1		0.054
	<60	5.9 (3.7–8.1)		1.954	0.989–3.862	
Sex	Male	7.8 (5.0–10.6)	0.464			
	Female	5.9 (4.3–7.6)				
Disease extent	LAPC	9.0 (3.6–14.3)	0.160	1		0.964
	MPC	6.2 (4.5–7.9)		1.022	0.401–2.606	
CA 19-9, U/mL	≥37.0	6.3 (4.6–8.1)	0.369			
	<37.0	7.8 (5.1–10.6)				
Total bilirubin, mg/dL	>1.2	5.8 (5.1–6.6)	0.448			
	≤1.2	6.9 (5.3–8.6)				
Albumin, g/dL	≥3.3	Not reached	0.428			
	<3.3	6.5 (4.9–8.0)				
sPD-L1, ng/mL	≥4.6	4.1 (1.5–6.7)	0.021	2.077	0.915–4.712	**0.080**
	<4.6	7.8 (5.3–10.3)		1		
NLR	≥1.83	6.2 (4.8–7.6)	0.008	3.141	0.950–10.391	**0.061**
	<1.83	Not reached		1		
PLR	≥109.6	6.2 (5.2–7.3)	0.011	1.060	0.392–2.866	0.908
	<109.6	10.5 (0.1–22.3)		1		

Abbreviations: LAPC = locally advanced pancreatic cancer; MPC = metastatic pancreatic cancer; CA 19-9 = cancer antigen 19-9; sPD-L1 = soluble programmed death-ligand 1; NLR = neutrophil-to-lymphocyte ratio; PLR = platelet-to-lymphocyte ratio; mPFS = median progression-free survival; CI = confidence interval; and HR = hazard ratio.

In a univariate analysis for OS, the prognostic factors indicative of improved OS were older age (≥60 years), a low sPD-L1 level (<4.6 ng/mL), and a low NLR (<1.83) (Table 3; Fig. 1B). In a multivariate analysis, a low sPD-L1 level (HR, 2.796, 95% CI, 1.221–6.400, *P* = 0.015) and low NLR (HR, 4.823, 95% CI, 1.554–14.971, *P* = 0.006) were independent prognostic factors for prolonged OS.

**Table 3:** Univariate and multivariate Cox regression analyses for overall survival.

Variable		Univariate analysis		Multivariate analysis		
		mOS (95% CI) (months)	*P*-value	HR	95% CI	*P*-value
Age, years	≥60	17.1 (0.3–33.9)	0.029	1		0.087
	<60	10.3 (8.8–11.8)		1.878	0.913–3.865	
Sex	Male	12.6 (8.2–17.0)	0.514			
	Female	10.3 (8.3–12.3)				
Disease extent	LAPC	16.8 (16.2–17.4)	0.051	1		0.465
	MPC	10.0 (9.0–11.0)		1.395	0.572–3.402	
CA 19-9, U/mL	≥37.0	10.0 (8.8–11.2)	0.587			
	<37.0	16.7 (10.2–23.2)				
Total bilirubin, mg/dL	>1.2	9.7 (6.6–12.8)	0.255			
	≤1.2	11.4 (8.2–14.6)				
Albumin, g/dL	≥3.3	10.6 (8.7–12.5)	0.802			
	<3.3	9.9 (0.1–25.7)				
sPD-L1, ng/mL	≥4.6	8.0 (6.6–9.4)	0.003	2.796	1.221–6.400	**0.015**
	<4.6	12.6 (9.1–16.1)		1		
NLR	≥1.83	10.0 (8.8–11.2)	0.006	4.823	1.554–14.971	**0.006**
	<1.83	Not reached		1		
PLR	≥109.6	10.3 (8.4–12.2)	0.129	1.855	0.729–4.720	0.195
	<109.6	17.1 (3.7–30.5)		1		

Abbreviations: LAPC = locally advanced pancreatic cancer; MPC = metastatic pancreatic cancer; CA 19-9 = cancer antigen 19-9; sPD-L1 = soluble programmed death-ligand 1; NLR = neutrophil-to-lymphocyte ratio; PLR = platelet-to-lymphocyte ratio; mOS = median overall survival; CI = confidence interval; and HR = hazard ratio.

In patients diagnosed with metastatic pancreatic cancer, only a low sPD-L1 level was an independent factor for prolonged OS in univariate and multivariate analyses (8.4 months in patients with high sPD-L1 *vs*. 10.2 months in patients with low sPD-L1, *P* = 0.028 for the univariate analysis, Fig. 1C; HR, 3.249, 95% CI, 1.302–8.108, *P* = 0.012 for the multivariate analysis).

### Prediction of treatment response and the dynamics of sPD-L1 during chemotherapy

Among the 60 patients, blood samples were collected from all 60 patients at the time of initial diagnosis with unresectable pancreatic cancer, from 53 patients at the first response assessment time point, and from 25 patients at the time of disease progression. Three paired samples (collected at the diagnosis, first response assessment, and disease progression time points) were obtained from 25 patients.

When we evaluated the role of sPD-L1 in predicting treatment response during FOLFIRINOX chemotherapy, the sPD-L1 levels at diagnosis (prechemotherapy) could not predict the best ORR in a univariate analysis (*P* = 1.000; Table 4). However, older age (≥60) (13/25 [52%] for older age *vs*. 9/35 [25.7%] for younger age, *P* = 0.037; HR, 4.858, 95% CI, 1.344–17.563*, P* = 0.016) and a decreased sPD-L1 level at the first response assessment time point (postchemotherapy) compared with the initial diagnosis time point (prechemotherapy) (5/24 [20.8%] for increased sPD-L1 *vs*. 14/29 [48.3%] for decreased sPD-L1, *P* = 0.038; HR, 4.267, 95% CI, 1.123–16.212, *P* = 0.033) were predictive factors for the achievement of a best overall response (CR + PR) during FOLFIRINOX chemotherapy.

**Table 4:** Univariate and multivariate analyses of the overall response rate.

Characteristic		Best ORR (CR + PR)		*P*-value	Multivariate analysis		
		Achieved	Not achieved		OR	95% CI	*P*-value
Age, years	≥60	13 (59.1)	12 (31.6)	0.037	1		**0.016**
	<60	9 (40.9)	26 (68.4)		4.858	1.344–17.563	
Sex	Male	12 (54.5)	21 (55.3)	0.957			
	Female	10 (45.5)	17 (44.7)				
Disease extent	LAPC	4 (18.2)	10 (26.3)	0.542			
	MPC	18 (81.8)	28 (73.7)				
CA 19-9, U/mL	≥37.0	16 (72.7)	31 (81.6)	0.423			
	<37.0	6 (27.3)	7 (18.4)				
Total bilirubin, mg/dL	>1.2	5 (22.7)	7 (18.4)	0.688			
	≤1.2	17 (77.3)	31 (81.6)				
Albumin, g/dL	≥3.3	21 (95.5)	36 (94.7)	1.000			
	<3.3	1 (4.5)	2 (5.3)				
sPD-L1, ng/mL	≥4.6	3 (13.6)	5 (13.2)	1.000			
	<4.6	19 (86.4)	33 (86.8)				
Δ sPD-L1, ng/mL	≥0	5 (26.3)	19 (55.9)	0.038	4.267	1.123–16.212	**0.033**
(response-initial)	<0	14 (73.7)	15 (44.1)		1		
NLR	≥1.83	16 (72.7)	30 (78.9)	0.583			
	<1.83	6 (27.3)	8 (21.1)				
PLR	≥109.6	16 (72.7)	27 (71.1)	0.890			
	<109.6	6 (27.3)	11 (28.9)				

Abbreviations: ORR = overall response rate; CR = complete response; PR = partial response; LAPC = locally advanced pancreatic cancer; MPC = metastatic pancreatic cancer; CA 19-9 = cancer antigen 19-9; sPD-L1 = soluble programmed death-ligand 1; Δ sPD-L1 = the difference in sPD-L1 levels between the first response assessment time point and time of initial diagnosis with unresectable pancreatic cancer; NLR = neutrophil-to-lymphocyte ratio; PLR = platelet-to-lymphocyte ratio; OR = odd ratio; and CI = confidence interval.

Similar results were observed in patients with metastatic pancreatic cancer. Older age (10/16 [62.5%] for older age *vs*. 8/30 [26.7%] for younger age, *P* = 0.027; HR, 9.331, 95% CI, 1.644–52.950, *P* = 0.012) and decreased sPD-L1 after chemotherapy (3/17 [17.6%] for increased sPD-L1 *vs*. 12/23 [52.2%] for decreased sPD-L1, *P* = 0.046; HR, 8.172, 95% CI, 1.328–50.305, *P* = 0.023) were predictive factors for the achievement of a best overall response.

**Figure 2:**
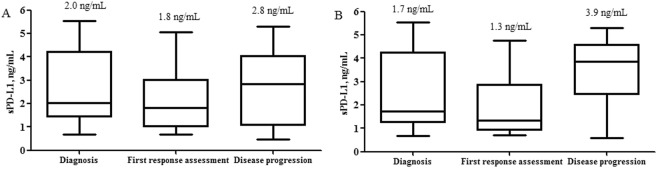
Comparison of soluble programmed death-ligand 1 (sPD-L1) levels among pancreatic cancer patients during FOLFIRINOX chemotherapy. (**A**) Time of diagnosis *vs*. first response assessment time point *vs*. disease progression time point (mean 2.6 ng/mL *vs*. 2.3 ng/mL *vs*. 2.6 ng/mL, respectively, *P* = 0.436) (**B**) Time of diagnosis *vs*. first response assessment time point *vs*. disease progression time point in patients who achieved complete response or partial response during chemotherapy (mean 2.5 ng/mL *vs*. 1.9 ng/mL *vs*. 3.5 ng/mL, respectively, *P* = 0.006).

When we compared the three paired samples, which were obtained at the diagnosis, first response assessment, and disease progression time points (n = 25) during FOLFIRINOX chemotherapy, sPD-L1 levels were decreased at the first response assessment time point compared to the time of diagnosis. However, this change was not statistically significant (median 2.0 ng/mL *vs*. 1.8 ng/mL; mean 2.6 ng/mL *vs*. 2.3 ng/mL, *P* = 0.254; Fig. 2A). The level of sPD-L1 at the time of disease progression was higher than that at the first response assessment time point, although this difference also failed to reach the level of statistical significance (median 1.8 ng/mL *vs*. 2.8 ng/mL; mean 2.3 ng/mL *vs*. 2.6 ng/mL, *P* = 0.394; total *P* = 0.436 among three paired samples; Fig. 2A).

In patients who achieved a CR or PR as their best response during chemotherapy, sPD-L1 levels tended to be decreased at the first response assessment time point compared with the time of diagnosis, although this difference was not statistically significant (n = 10; median 1.7 ng/mL *vs*. 1.3 ng/mL; mean 2.5 ng/mL *vs*. 1.9 ng/mL, *P* = 0.331; Fig. 2B). However, the level of sPD-L1 was significantly increased at the time of disease progression compared with the first response assessment time point (n = 10; median 1.3 *vs*. 3.9 ng/mL; mean 1.9 ng/mL *vs*. 3.5 ng/mL, *P* = 0.025; total *P* = 0.006 among three paired samples; Fig. 2B).

## Discussion

In this study, pancreatic cancer patients with high sPD-L1 levels at the time of diagnosis showed significantly worse OS than patients with low sPD-L1 levels despite identical FOLFIRINOX chemotherapy treatment. Furthermore, patients whose sPD-L1 levels decreased between the time of diagnosis (prechemotherapy) and the first response assessment time point showed better responses to FOLFIRINOX than those whose sPD-L1 levels increased after chemotherapy.

Pancreatic cancer is a highly lethal malignancy worldwide^17,18^. Although recent palliative chemotherapies have improved the median OS of metastatic pancreatic cancer patients up to 8.5–11.1 months, the efficacy of current treatment options is still very limited^19,20^. In the era of immunotherapy, large randomized clinical trials have reported promising outcomes in patients with various cancers, such as lung cancer, melanoma, and renal cell carcinoma^21–23^. However, in pancreatic cancer, clinical trials of anti-PD-L1 monotherapies have shown disappointing outcomes^13,24^. The reason for this failure could be partly explained by the nonimmunogenic tumor microenvironment of pancreatic cancer^25^. The efficacy of immune checkpoint blockade has been related to the expression of PD-L1 by tumor cells and PD-1 by activated T cells. However, a previous study revealed that pancreatic cancer had low expression of both PD-1 and PD-L1^26^. In addition, although patients have high levels of PD-L1 expression, highly immunosuppressive microenvironment elements, such as regulatory T cells, myeloid-derived suppressor cells and tumor-associated macrophages, might affect the unsuccessful treatment response to immunotherapy^25^.

Due to its nonimmunogenic nature, pancreatic cancer has not yet been evaluated in a way that fully elucidates the prognostic role of PD-L1. It has been suggested that an immune checkpoint might not entirely represent the histopathological hallmarks of this malignancy^25,27^. However, several studies have reported a prognostic role for PD-L1. *Tessier-Cloutier et al*. demonstrated that increased PD-L1 expression (>10%) detected by immunohistochemistry was associated with poor disease-specific survival in resected pancreatic cancer patients (median 0.61 years *vs*. 1.52 years, *P* = 0.027)^11^. *Yamaki et al*. revealed that PD-L1-positive patients showed worse OS than PD-L1-negative patients who underwent surgical resection (HR, 2.07, 95% CI, 1.00–4.54; *P* = 0.049)^12^. However, in an analysis using sPD-L1, *Kruger et al*. reported that sPD-L1 did not predict adverse outcomes in patients with advanced pancreatic cancer (11.92 months for high sPD-L1-expressing patients *vs*. 9.53 months for low sPD-L1-expressing patients, *P* = 0.36). They found that the levels of sPD-L1 were increased in patients with elevated C-reactive protein levels (*P* < 0.001), suggesting that sPD-L1 could be a marker of systemic inflammation in advanced pancreatic cancer^28^.

In this study, we also demonstrated the dynamics of sPD-L1 during homogenous FOLFIRINOX chemotherapy. sPD-L1 levels at the first response assessment time points were lower than those at the time of diagnosis. sPD-L1 levels were also higher at the disease progression time point than at the first response assessment time point. Although this study did not achieve statistical significance due to the small sample size, the observed changes in the sPD-L1 levels according to disease status were clinically important. In patients who achieved a CR or PR as their overall response, the sPDL1 levels were significantly increased at the disease progression time point compared to the first response assessment time point. Therefore, dynamic changes in sPD-L1 levels during chemotherapy correlated with disease progression.

Although inflammatory markers such as the NLR and PLR did not correlate with sPD-L1 levels in our study, elevated NLR levels were associated with poor OS. This finding was in accordance with the results of previous studies that showed a relationship between increased systemic inflammation and poor outcomes in pancreatic cancer^29,30^.

The present study has several limitations. It was a single-center study, and the sample size was small. Therefore, further large-scale studies are needed to confirm our results. We enrolled only advanced-stage pancreatic cancer patients who were treated with FOLFIRINOX chemotherapy as a palliative first-line treatment to focus on a relatively homogeneous population. Patients who received other standard of care treatments, such as gemcitabine/nab-paclitaxel combination therapy or gemcitabine monotherapy, were excluded. A study of pancreatic cancer patients treated with these standard regimens is warranted to further support our conclusion for advanced pancreatic cancer. Despite these limitations, to the best of our knowledge, this is the first study to show the dynamics of sPD-L1 levels in unresectable pancreatic cancer patients treated with homogenous FOLFIRINOX chemotherapy. This study provides evidence that the prechemotherapy sPD-L1 level could be a prognostic factor for OS and that the dynamics of sPD-L1 levels during chemotherapy could predict treatment responses. In routine clinical practice, sPD-L1 measurement in patient blood samples could be easily incorporated. However, the standardization of this measurement should be further validated beforehand.

## Conclusions

In conclusion, pretreatment sPD-L1 levels play a significant role in predicting survival outcomes in advanced pancreatic cancer patients treated with FOLFIRINOX chemotherapy. Additionally, the dynamics of sPD-L1 levels during chemotherapy could be used to predict the best treatment response during chemotherapy.

## Data Availability

The datasets used in the current study are available from the corresponding author on reasonable request.
